# The immediate insertion, loading and provisional prosthetic restoration of dental implants

**Published:** 2008-04-15

**Authors:** Sârbu I

**Affiliations:** * “Carol Davila” University of Medicine and Pharmacy, Bucharest, Romania

**Keywords:** immediate implant loading, bone implant interface, guided tissue regeneration, structural biology, tissue regeneration physiology, early implant loading, osseointegration

## Abstract

The immediate insertion and loading of dental implants although used in the past as an alternative method for the surgical phase of the dental implant treatment is today becoming more and more popular due to its immediate and spectacular results.

With information on bone density and a careful patient selection, this method has increased chances of success. Its main advantage is the high degree of patient comfort with a great aesthetic effect.

This article presents the theoretical and practical technique used on two clinical cases of dental implantation and their outcome.

Today the main goal of the modern dentistry is to enhance the comfort of the patient during treatment and also during the intermediate phases, at the same time shortening the total treatment time. Many times, long waiting periods, especially in oral implantology, are a great stress factor for both the patient and the doctor. 

In the past, the surgical phase of the dental implant treatment was composed of two interventions, to offer the implant a healing period with no occlusal load and away from the septic oral environment. The immediate loading of dental implants was initially an alternative method, however, in time it became more popular among practitioners thanks to the studies that showed no difference histologically at the bone-implant interface between an immediate and a tardive loaded implant. The immediate mounting of a prosthetic abutment and the immediate provision at the surgical phase allow the patient to get over the psychological trauma of the edentulous state, especially when we refer to losing a tooth in the frontal maxillary area. 

This method gives the patient more trust in both the treatment and the doctor because the result of the surgical intervention is immediate and spectacular.

The limitations of the method are given by the factors which prohibit the immediate loading of a dental implant – important bone defects which require augmentation, low-density bone and poor primary stabilization of the implant. 

As dental implants have become a more common, predictable and financially approachable clinical procedure for the edentulous (partially or completely) in order to maintain a low degree of failures, it is of extreme importance to make a perfect diagnosis, develop a good treatment plan and perform the most perfect clinical procedures. Having the information regarding the bone density and making a good patient selection further improves the chances of treatment success.

The bone density is required to be assessed pre-operatively, by an osteodensitometric exam or by cat-scan. The bone density measurements are very important in determining the primary stability – the lack of pathological implant movement immediately after placement. If the bone has a low density we prefer to perform a bone compaction before inserting the implant and to load it after 4-6 months. 

To avoid creating bone defects of the extractional socket, the extraction must be performed with minimal trauma, without forcing the buccal or oral plates and without fracturing them. Ideally, before beginning tooth removal we recommend to perform the sindesmotomy with a piezotome, this way ensuring the cortical plates are fully conserved. 

The primary stability of the inserted implant is assessed with the dynamometric ratchet. A dental implant is considered to be able to resist an immediate load with a torque greater than 35 Ncm. Also the “Periotest” device measures the breaking point when tapping the implant surface and it offers valuable information on the primary stability of an implant, considering it very stable if the value shown is between -3 and -9. 

Placing an implant into a fresh alveolus will usually result in a gap between the implant and the bone walls. To ensure proper conditions for optimal healing, synthetic bone substitutes, membranes, bone grafting, osteoinductive substances or a combination of these must be used to determine the formation of bone tissue around the implant. 

The method we propose for the immediate provision on an immediately loaded post extractional implant uses the natural crown of the extracted tooth, provided it does not have cavities or big restorations. 

The main advantage of the method is the high degree of comfort the patient has immediately after surgery, because basically he does not feel he had lost a tooth, having his own natural crown attached to the implant. The aesthetic effect is maximum, and the patient can integrate back into the society immediately after surgery, without any modifications in his smile. From the practitioner’s point of view, the method is very simple, it does not require special materials or devices, and is performed quicker than when using a standard acrylic provisional because the final restoration does not need shaping and polishing.

We present the method in a couple of clinical cases:

Clinical case # 1

**Fig. 1:**
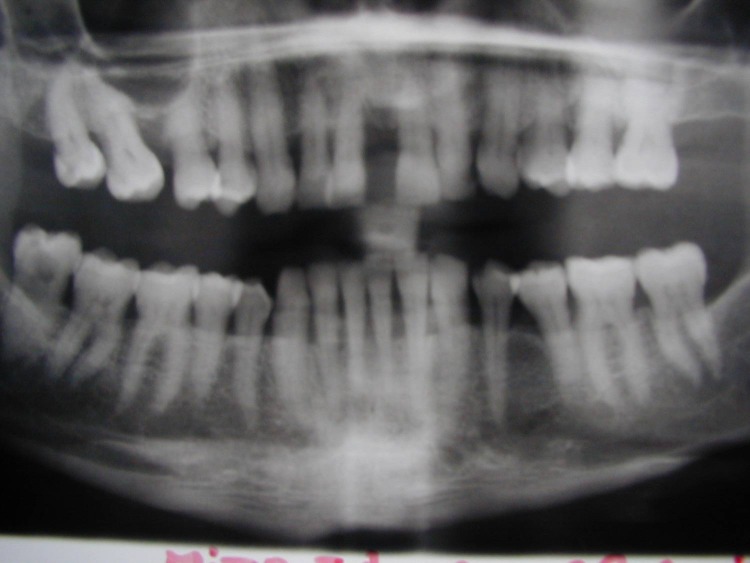
Pre-op x-ray

**Fig. 2:**
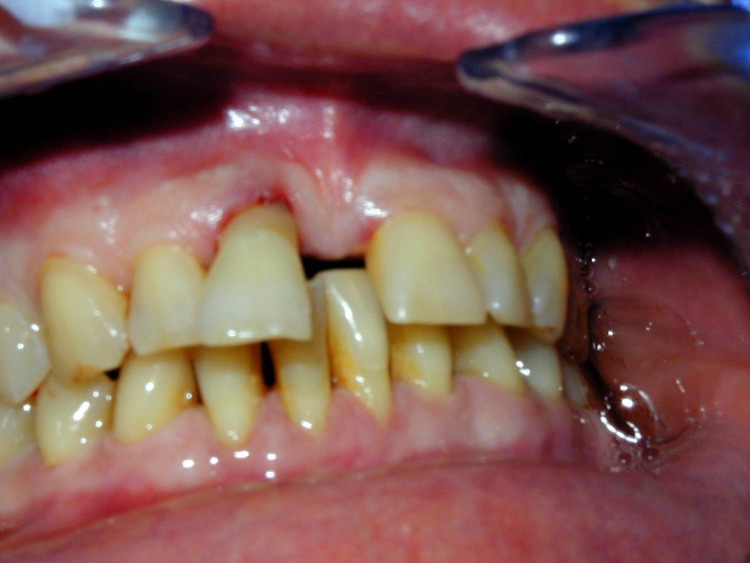
Pre-op oral aspect. The patient accuses pain and mobility of tooth 1.1.

**Fig. 3:**
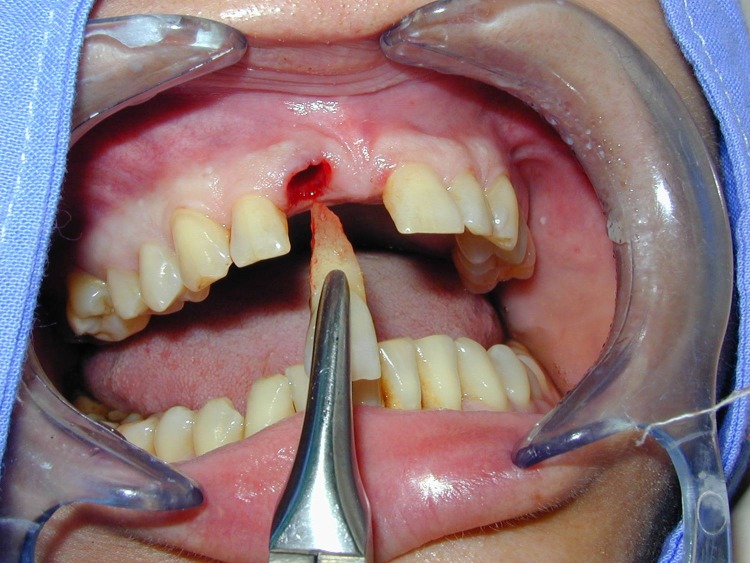
We decided to extract 1.1 (as atraumatic as possible) and to insert immediately postextractional a dental implant

**Fig. 4:**
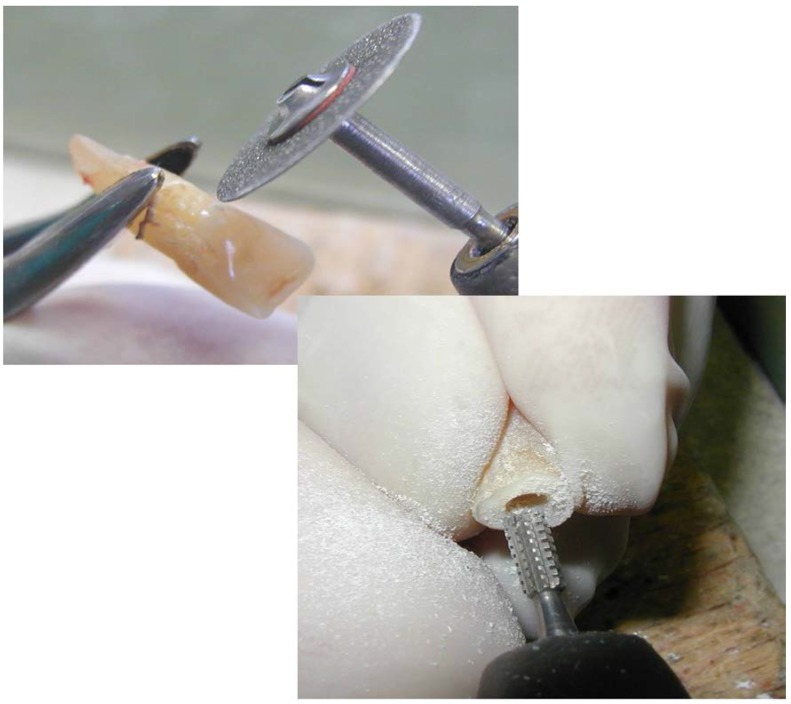
The crown of 1.1 is separated from the root and is drilled to fit on the prosthetic abutment of the implant

**Fig. 5:**
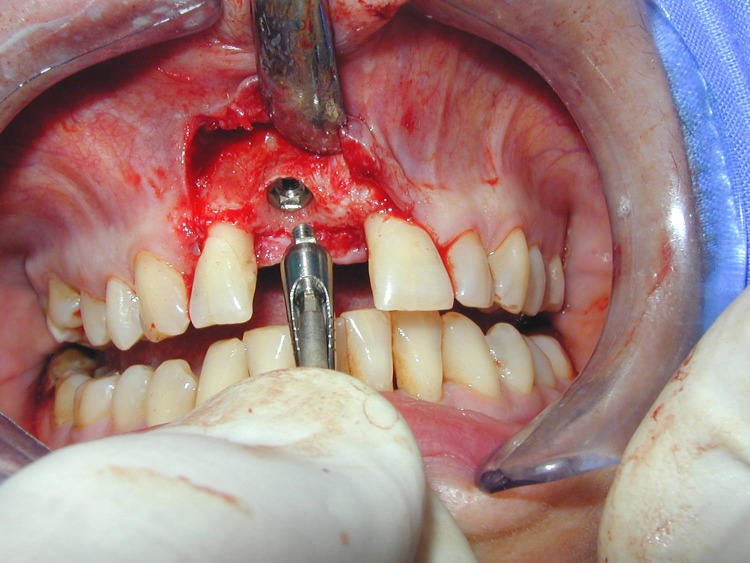
After seating the implant the prosthetic abutment is mounted, before suture.

**Fig. 6:**
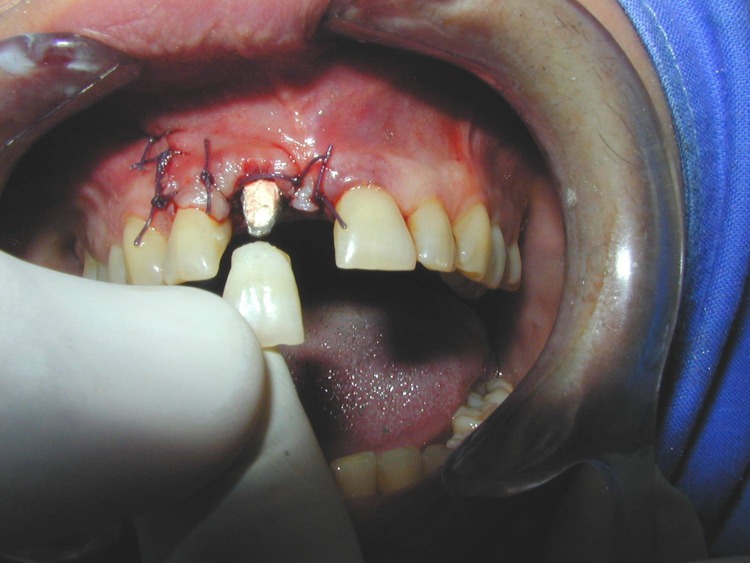
Try-in of the crown on the prosthetic abutment.

**Fig. 7:**
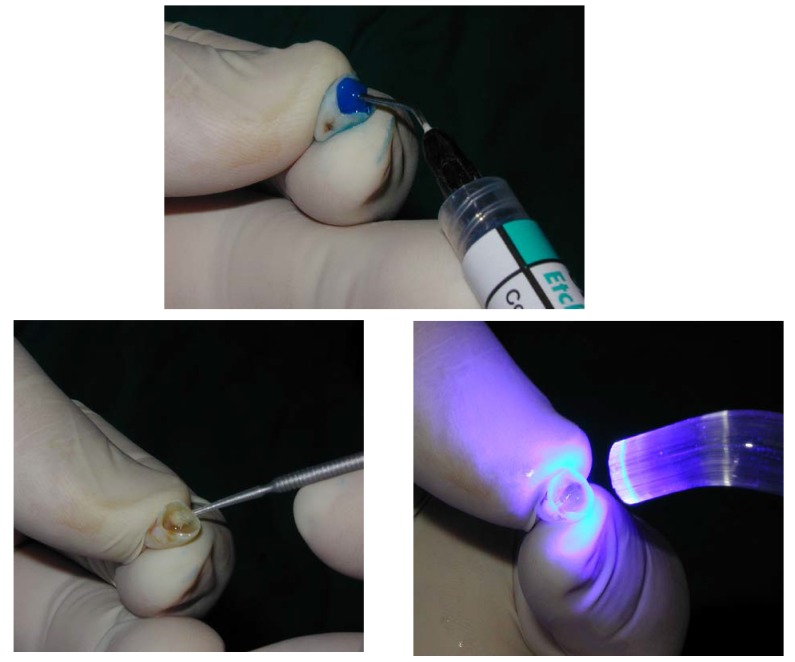
After the try-in the inside of the crown is etched and a thin layer of bonding is applied.

**Fig. 8:**
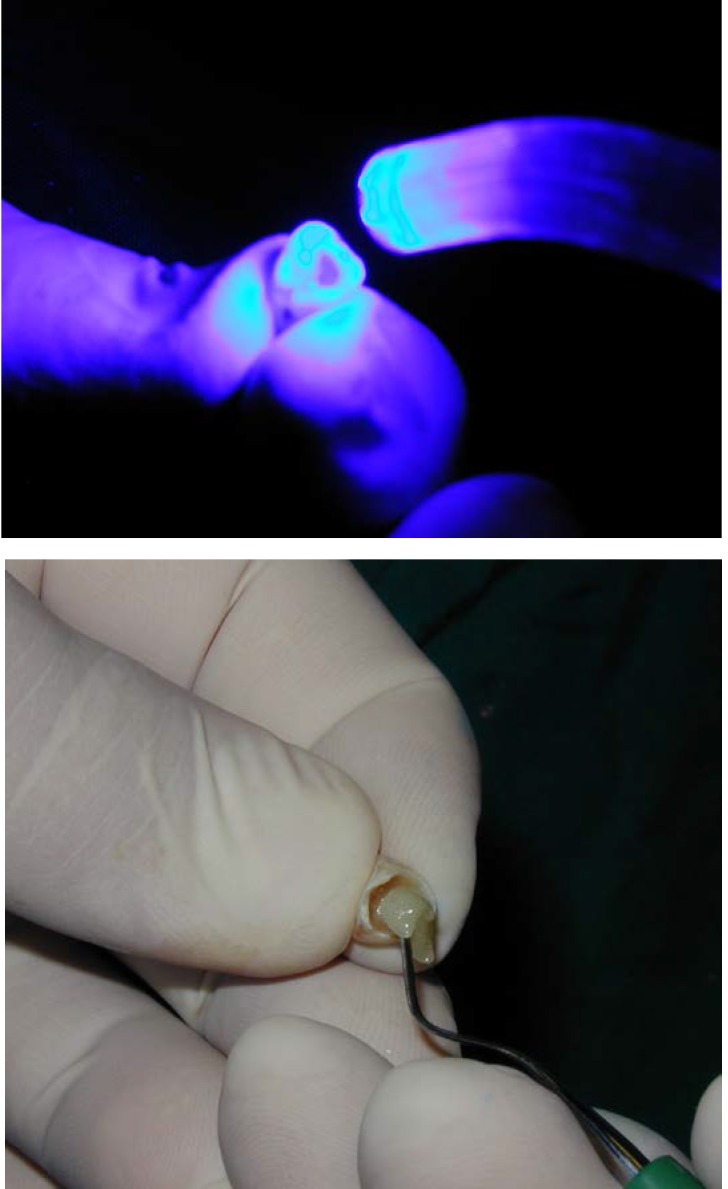
The crown is filled with light-curable composite and then it’s seated on the prosthetic abutment. The crown is then carefully removed from the prosthetic abutment, and the light-curing of the composite (started while it was placed on the abutment) is finished

**Fig. 9:**
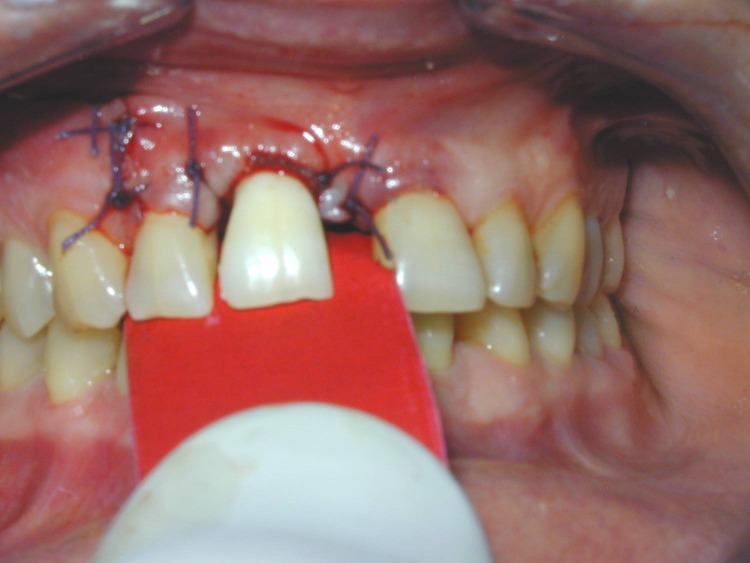
Occlusion check. The crown has no occlusal contacts.

**Fig. 10:**
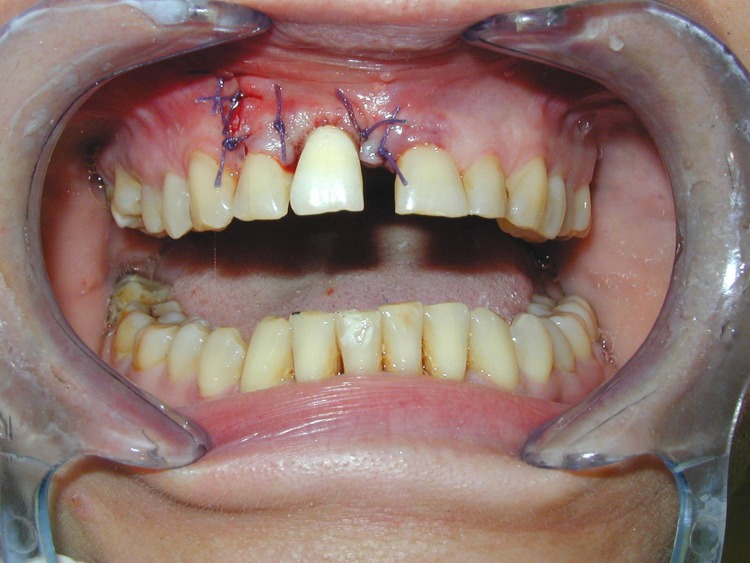
Intraoral view immediately post-operative

**Fig. 11:**
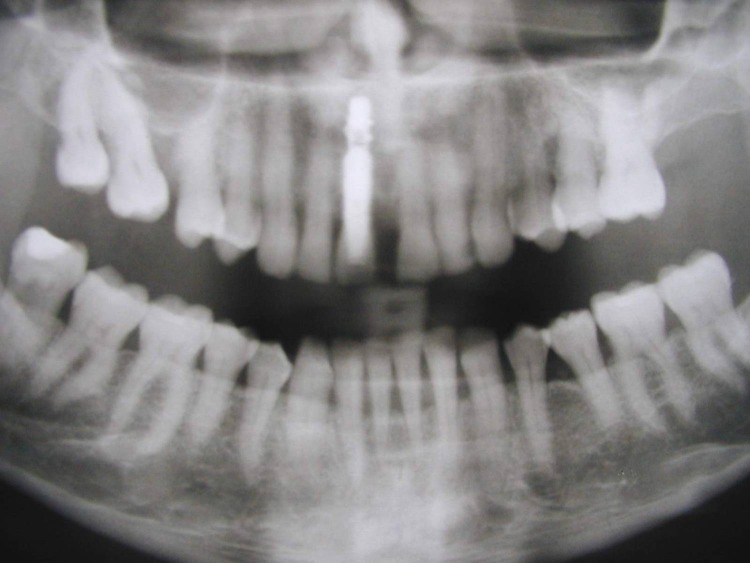
Post-op x-ray

**Fig. 12:**
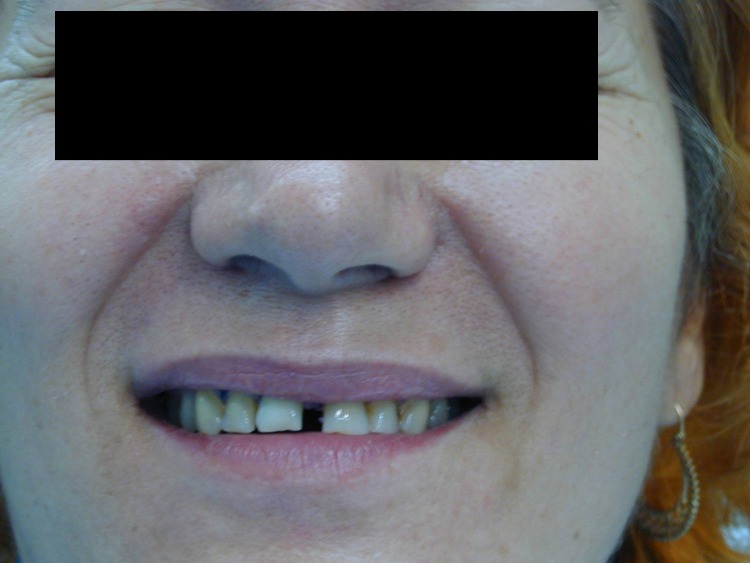
The patient before leaving the practice, immediately after surgery.

Clinical case #2.

**Fig. 13:**
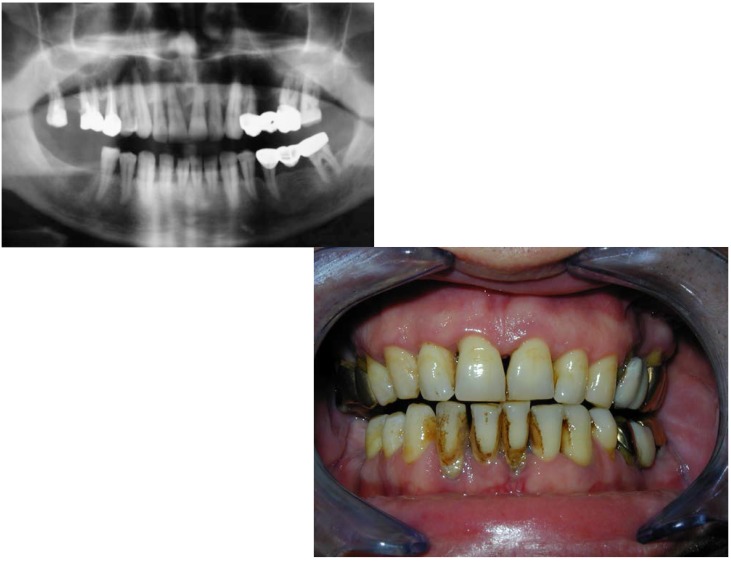
Preop x-ray and intraoral view of a 47 years old patient. He accused pain and mobility of tooth 1.1. Before the surgery a throughout cleaning is performed.

**Fig. 14:**
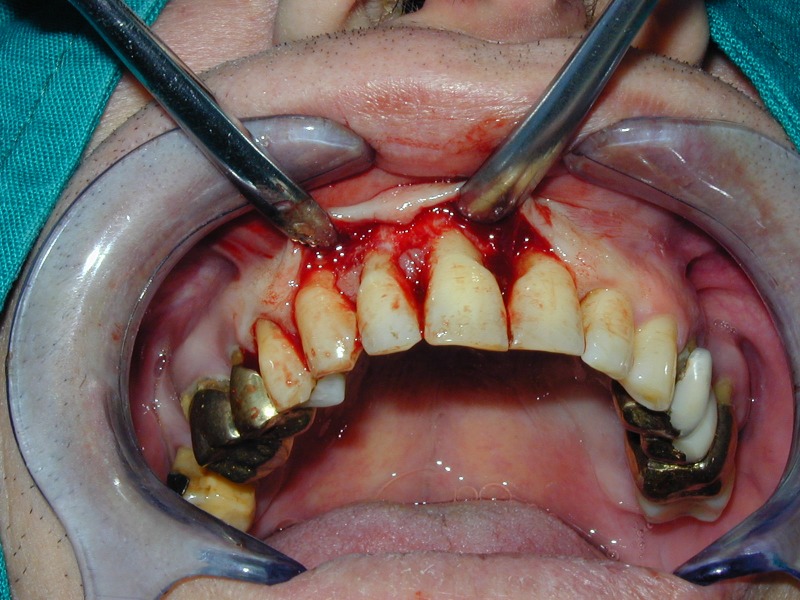
Reflection of the buccal flap.

**Fig. 15:**
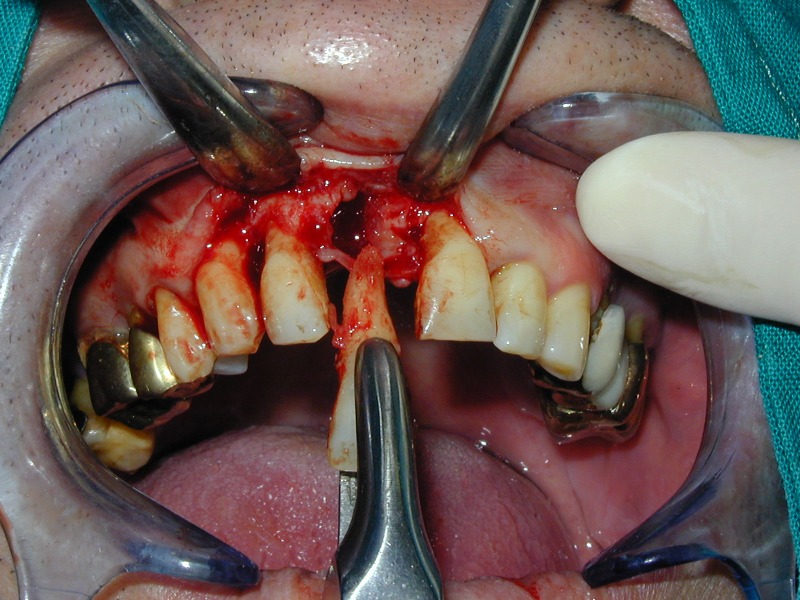
Atraumatic extraction of 1.1.

**Fig. 16:**
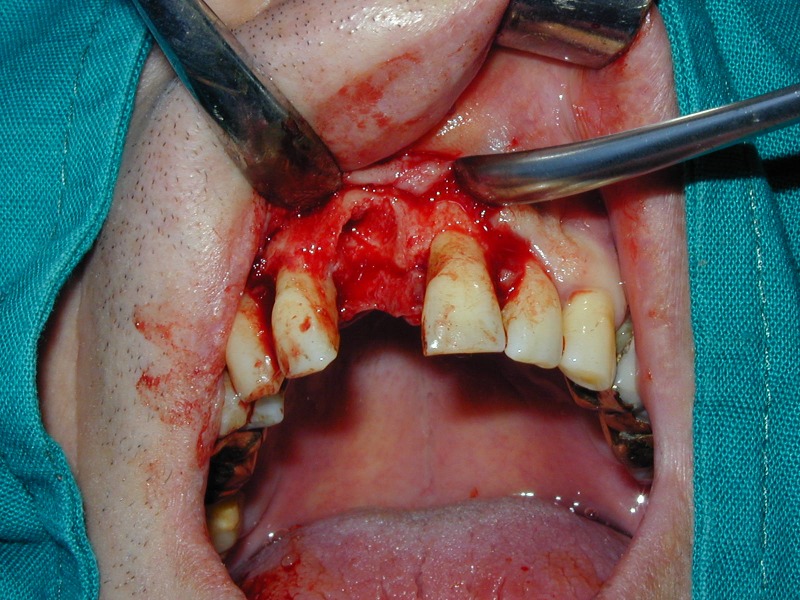
Extractional socket after removing the granulation tissue.

**Fig. 17:**
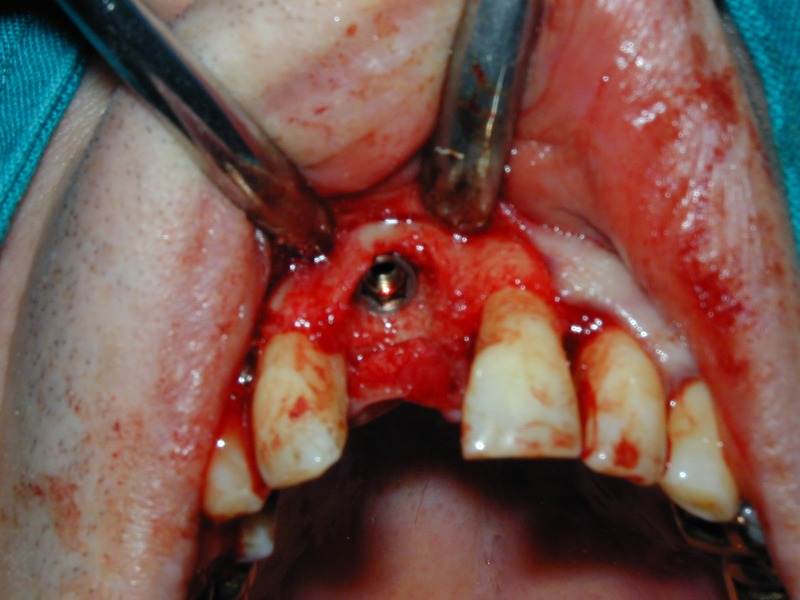
The implant after seating.

**Fig. 18:**
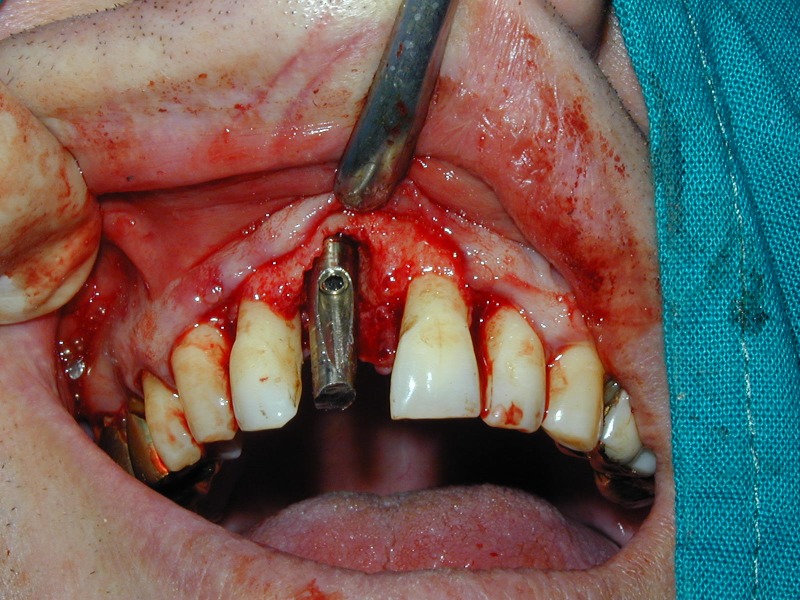
Mounting of the prosthetic abutment.

**Fig. 19:**
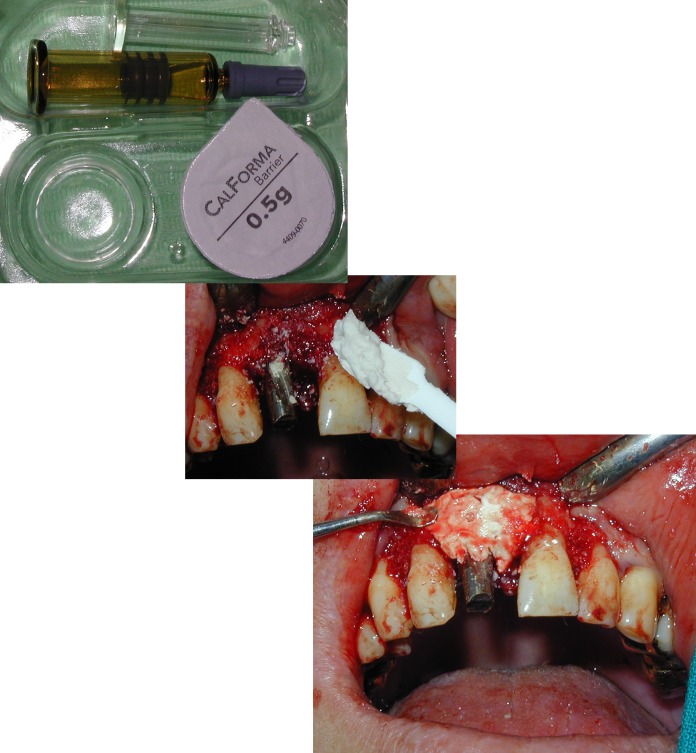
The buccal bony defect is protected with a calcium sulfate barrier after augmentation

**Fig. 20:**
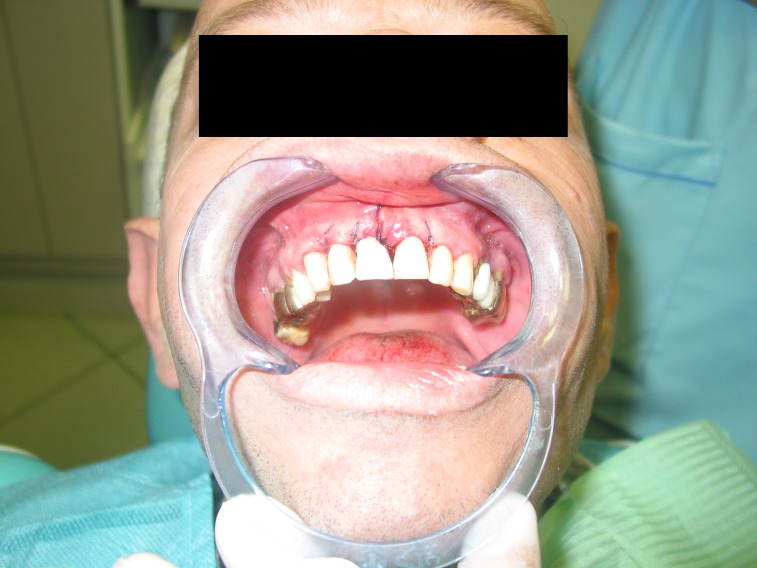
Intraoral aspect immediately after surgery.

**Fig. 21:**
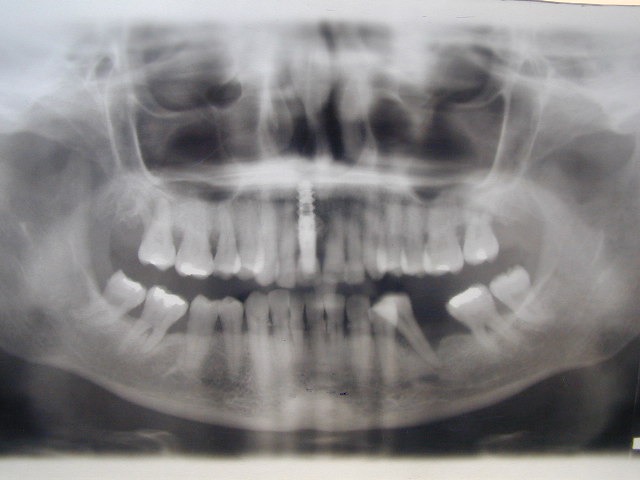
Post-op x-ray.

**Fig. 22:**
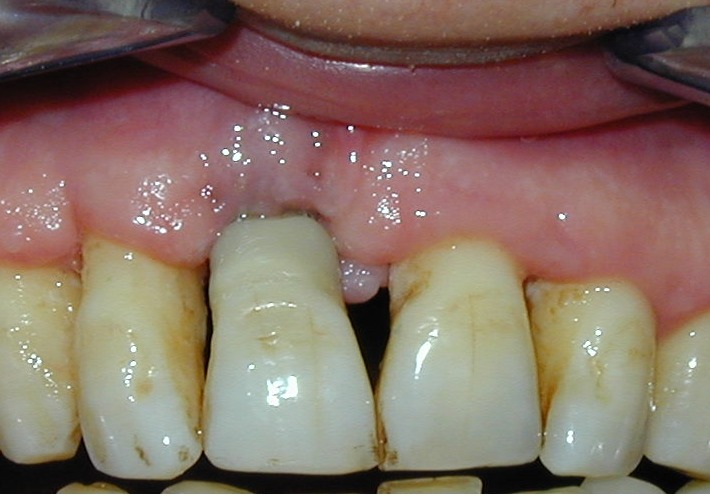
Intraoral image 6 months after surgery.

